# FBA-PRCC. Partial Rank Correlation Coefficient (PRCC) Global Sensitivity Analysis (GSA) in Application to Constraint-Based Models

**DOI:** 10.3390/biom13030500

**Published:** 2023-03-09

**Authors:** Anatoly Sorokin, Igor Goryanin

**Affiliations:** 1 Okinawa Institute of Science and Technology Graduate University, Okinawa 904-0495, Japan; 2 Tianjin Institute of Industrial Biotechnology, Chinese Academy of Sciences, Tianjin 300308, China; 3 School of Informatics, The University of Edinburgh, Informatics Forum, Edinburgh EH8 9AB, UK

**Keywords:** whole-genome model, constraint-based model, metabolic network, global sensitivity analysis, FBA

## Abstract

Background: Whole-genome models (GEMs) have become a versatile tool for systems biology, biotechnology, and medicine. GEMs created by automatic and semi-automatic approaches contain a lot of redundant reactions. At the same time, the nonlinearity of the model makes it difficult to evaluate the significance of the reaction for cell growth or metabolite production. Methods: We propose a new way to apply the global sensitivity analysis (GSA) to GEMs in a straightforward parallelizable fashion. Results: We have shown that Partial Rank Correlation Coefficient (PRCC) captures key steps in the metabolic network despite the network distance from the product synthesis reaction. Conclusions: FBA-PRCC is a fast, interpretable, and reliable metric to identify the sign and magnitude of the reaction contribution to various cellular functions.

## 1. Introduction

Genome-scale metabolic models (GEMs) that combine functional annotation of the genome with available metabolic knowledge are an valuable tool for modern computational and systems biology [1,2]. GEMs were used in biotechnology for strain engineering [3,4,5] for a better understanding of the metabolic consequences of various pathological processes [6,7,8], such as cancer [9,10], metabolic syndrome, and obesity [11], to name a few. Over the past decade, GEMs have been created for several hundreds of unicellular organisms (BiGG [12], MEMOTE [13], AGORA [14]) and dozens of human body tissue types [15]. However, most of these models were created by either semi- or fully-automated processes, which could suffer from incorrect gene annotation, arbitrary reactions added by the gap-filling process [13,14,16], etc. All of these inflate the size of the model, tangle it with unnecessary reactions, and make its analysis more complicated. There is a quote attributed to Einstein: “You should make things as simple as possible, but not simpler”. The model reduction methods applicable to the GEMs are an active area of research [17,18]. Here we propose another approach to model simplification, which is based on flux balance analysis (FBA) and global sensitivity analysis (GSA).

FBA is a computational approach used to analyze and predict the metabolic behavior of an organism using GEM [19]. FBA is commonly used in systems biology and metabolic engineering to study cellular metabolism and to predict the growth and behavior of an organism in different conditions. FBA is based on the principle of mass balance, where the input and output of each metabolite in a metabolic network are balanced. The metabolic network is represented as a set of reactions, which are connected by metabolites. Each reaction has an associated flux, which represents the rate at which the reaction occurs. FBA uses linear programming to optimize the flux through the metabolic network of GEM, subject to constraints such as the availability of nutrients and the capacity of enzymes. The goal of FBA is to find the set of fluxes that maximizes a specific objective function, such as the growth rate of the organism. The FBA approach can be used to predict the effect of genetic and environmental perturbations on cellular metabolism, and to identify metabolic engineering targets for improving the performance of industrial bioprocesses. FBA has been successfully applied to a wide range of organisms, including bacteria, yeast, plants, and humans.

Kinetic modeling involves creating mathematical models that describe the behavior of complex systems, such as chemical reactions, biological processes, or ecological systems. These models often contain a large number of parameters, each representing a specific aspect of the system, such as reaction rates, enzyme concentrations, or external inputs. However, not all of these parameters are equally important for the behavior of the system. Some parameters may have little influence on the system trajectory, and their values may be difficult or impossible to estimate from experimental data. These unimportant parameters are called unobservable parameters. Identifying unobservable parameters is important because it can help simplify the model and reduce the number of unknowns that need to be estimated. One approach to identifying unobservable parameters is global sensitivity analysis (GSA), which estimates how variations in each parameter value affect the behavior of the system as a whole [20]. GSA can help identify parameters that have little influence on the system behavior, and therefore can be considered unobservable.

One approach to identifying unobservable parameters in FBA is Flux Variability Analysis (FVA). FVA estimates the range of possible flux values for each reaction in the network, while keeping the objective near its optimal value. This allows researchers to identify reactions that are essential for the network’s behavior, which have narrow flux distribution, as well as reactions that can be varied without significantly affecting the network’s output. However, FVA does not provide information about flux interactions and how fluxes influence the objective value when it is far from optimum, which can be important for understanding the behavior of complex networks. To analyze flux interactions, Kelk and colleagues have developed a method called CoPE-FBA [21], which utilizes a decomposition approach to break down alternative flux distributions into three topological features: vertices, rays, and linealities. These features correspond to paths, irreversible cycles, and reversible cycles in a metabolic network, respectively. The authors demonstrated that the optimal solution space is often determined by a few subnetworks or modules consisting of numerous reactions, each with multiple internal routes. By analyzing the solution space using this method, it is possible to characterize the entire space based on these subnetworks or modules. As a result, two reactions would be present in the same module if their flux values across all vertices are correlated, regardless of whether they are in the same flux route or in exclusive ones. To analyze how flux perturbation influences other fluxes and objectives, a local version of sensitivity analysis in FBA that is combining FVA with Monte-Carlo sampling was developed [22]. In this approach, all reactions are divided into three groups: ‘stable’ reactions have a low FVA range, ‘robust’ reactions vary a little with perturbations of the other reaction fluxes, and ‘sensitive’ reactions significantly change their fluxes in response to the perturbation. Then, the fraction of each group was compared between different constraint types and mutations. However, neither the CoPE-FBA nor Monte-Carlo approach show how variation in the flux influences the objective value.

Recently, a global sensitivity analysis (GSA) of constraint-based models was published in the literature [23]. This type of analysis is useful for identifying which model parameters have the greatest impact on the model output, and for understanding the behavior of the model in response to changes in those parameters. However, the authors of the study chose to use a relatively complicated and computationally expensive method of GSA called Sobol variance-based sensitivity analysis.

Sobol variance-based sensitivity analysis is a powerful tool for quantifying the contribution of individual parameters and interactions between parameters to the variability of the model output. It is based on the decomposition of the variance of the model output into contributions from individual parameters, as well as combinations of parameters. This allows the authors to identify which parameters have the greatest impact on the output, and to quantify the degree to which the interactions between parameters affect the model behavior. This method of GSA is computationally expensive and requires the development of a complex computational infrastructure. This may limit its applicability in some contexts, particularly for models that are computationally intensive or have a large number of parameters. Moreover, it may require specialized expertise in order to implement and analyze the results of the method.

Despite its limitations, Sobol variance-based sensitivity analysis remains a powerful tool for GSA and can provide valuable insights into the behavior of complex models. It is important for researchers to carefully consider the trade-offs between computational cost and analytical power when selecting a method for GSA, and to choose a method that is well-suited to the specific needs of their study.

In response to the limitations of Sobol variance-based sensitivity analysis, a new approach has been proposed for estimating objective function sensitivity to flux boundary values using Partial Rank Correlation Coefficient (PRCC) calculations [20]. The PRCC approach is based on calculating the partial correlation coefficient between the ranks of each parameter and the rank of the objective function value:

(1) $$r_{x_{j},y}=\frac{Cov\left(\hat{x_{j}},\hat{y}\right)}{\sqrt{Var\left(\hat{x_{j}}\right)Var\left(\hat{y}\right)}};\;\hat{y}=y-\tilde{y};\;\hat{x_{j}}=x_{j}-\tilde{x_{j}}$$

where $\tilde{y}$ and $\tilde{x_{j}}$ are obtained from linear regression models:

(2) $$\tilde{x_{j}}=c_{0}+\sum_{p=1,p\neq j}^{N}c_{p}x_{p};\;\tilde{y}=b_{0}+\sum_{p=1,p\neq j}^{N}b_{p}x_{p}$$

This approach provides a measure of the sensitivity of the objective function to each parameter, while taking into account the interactions between parameters. Rank-transformed data are used to take into account possible nonlinearity in the data.

One advantage of the PRCC approach is that it does not require extensive coding and can be implemented using standard flux balance analysis (FBA) tools, such as the Cobrapy toolbox [24]. The calculation time for the PRCC approach depends on the number of available CPUs in the high-performance computing (HPC) cluster, as parallelization is applied at the level of flux boundaries. This approach is therefore computationally efficient and can be applied to large-scale models with many parameters.

Another benefit of the PRCC approach is that the sensitivity coefficient is signed, allowing researchers to distinguish between parameters that positively or negatively influence the objective function. This provides additional insight into the behavior of the model and can help guide the selection of interventions or modifications to the system.

To calculate the PRCC sensitivity coefficient, a set of random points in the parameter set is sampled using the Sobol low discrepancy sequence, as described in previous work [25,26,27,28]. The PRCC sensitivity coefficient is then calculated as a partial correlation coefficient between each parameter and the objective function value, with the influence of other parameters controlled for.

Marino and co-authors [20] provide methods to estimate both the significance and saturation of the PRCC sensitivity coefficient. The significance of the coefficient is determined by comparing its magnitude to the distribution of coefficients obtained from randomized permutations of the data. The saturation of the coefficient is a measure of how much of the variation in the objective function can be explained by the variation in the parameter value, with higher saturation indicating a stronger relationship between the parameter and the objective function.

In summary, the PRCC approach provides a computationally efficient and flexible alternative to Sobol variance-based sensitivity analysis for conducting GSA in constraint-based models. Its ease of implementation and ability to provide signed sensitivity coefficients make it a valuable tool for studying the behavior of complex systems.

## 2. Materials and Methods

The metabolic network with m metabolites and r reactions is described by an m·r stoichiometry matrix, N. The (i, j)-th entry of N, $n_{\mathrm{ij}}$, is the stoichiometric coefficient of the i-th metabolite in the j-th reaction. Any reaction flux vector v that satisfies Nv=0 contains reaction fluxes such that the system is in a steady state. In Flux Balance Analysis (FBA) [18], some optimization problem is solved to identify a unique solution vector $v_{o}$, such that $wv_{o}=\underset{v}{\max}wv$ for $v_{l}\leq v\leq v_{u}$, where *w* is the objective coefficient vector and $v_{l}$ and $v_{u}$ are reaction bounds. We are interested in the estimation of the sensitivity of the objective function to the values of reaction boundaries.

There is a special type of reaction in the constraint-based modelling called ‘boundary reactions’, which usually describe the exchange of metabolites between the internal ‘cell’ and the external ‘environment’.

Our approach consists of three steps:

1. Define parameter space: for non-boundary irreversible reactions only one parameter $v_{u}$ is created, for reversible and boundary reactions two parameters are created for each reaction$—v_{l}$ and $v_{u}$.
2. Generate a set of quasi-random low-discrepancy points in the parameter space. Update parameters (reaction bounds) and find the optimal objective value for each point in the parameter space.
3. Calculate Partial Rank Correlation Coefficient (PRCC) for each parameter and objective value. The statistical significance of the PRCC value is estimated as described by Marino et al. [20]. The sufficiency of the sample size for reliable PRCC estimation is controlled by the top-down coefficient of concordance (TDCC): when TDCC between PRCC vectors calculated at different sample sizes exceeds the threshold of 0.9, the sample size is considered sufficient for analysis.

**Figure 1:**
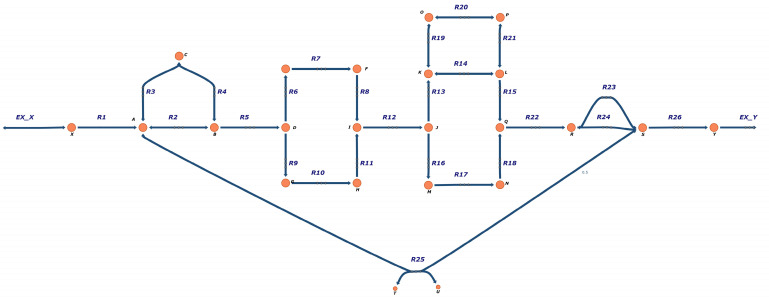
Toy metabolic model with 23 metabolites and 27 reactions. The source and sink metabolites are X, Y, T, and U, their concentrations are considered fixed in order to ensure a steady state, which we assume to be stable. Reversible reactions are depicted by two-way arrows, irreversible reactions by one-way arrows. FBA was applied to maximize the flux through reaction EX_Y.

The toy model (Figure 1) was created with Cobrapy v.0.25.0 [24] and saved as JSON for the model diagram drawing with Escher web interface [29] and SBML [30] format for further simulations. *E. coli* str. K-12 substr. W3110 WGMM was taken from BiGG database [12] in SBML format.

For network distance calculations, all metabolites participating in more than 20 reactions in any compartment, except amino acids, succinate, PEP, and fructose-6-phospate, were removed. Network distance was calculated as the number of reaction steps between the node of interest and objective reaction. Calculations were performed with R package ‘igraph’ version 1.3.5 [31].

The Sobol quasi-random low-discrepancy sequence was generated with the python Quasi-Monte Carlo submodule of the SciPy v.1.7.3 [32]. Model simulation was performed with Cobrapy v.0.25.0 [24]. All calculations were performed using Python version 3.10.2.

PRCC calculations were performed with R package ‘sensitivity’ version 1.28.0 [33]. TDCC values were calculated by ‘ODEsensitivity’ R package version 1.1.2 [34]. All calculations were performed with R version 4.2.1 [35].

All simulations were performed on the OIST HPC cluster with 8CPU and 64GB per job. Sobol points generation, application to the reaction boundaries and optimization of objectives were performed in chunks of 8192 per job. Calculations of the PRCC sensitivity coefficients were performed on 262,144 Sobol points in chunks of 10 features per job. Convergence of the calculation was controlled by TDCC between consecutive datasets different in 8192 Sobol points. The TDCC value between 262,144 and 253,952 was 0.909. The average execution time was 30 min per job for the Sobol point calculations and 7 h per job for the PRCC calculation.

## 3. Results

Techniques such as Flux Balance Analysis (FBA) utilize the stoichiometry matrix of the reaction system to estimate steady-state fluxes, which are compatible with the viable state of the system. In FBA, an objective is optimized over the steady-state flux vectors, usually by maximizing the flux through certain reactions. The behavior of constraint-based models is controlled by parameters, such as reaction flux boundaries. Our approach estimates the sensitivity of the model’s objective function to these boundary values.

### 3.1. FBA-PRCC Can Identify the Backbone of the Flux-Related Network

To construct the parameter space, we consider that reversible reactions have two boundaries, whereas irreversible reactions have only one, with the lower bound usually set to zero. Boundary reactions, which describe the transport of matter through the model boundary, require special treatment. To evaluate the sensitivity of the objective function to the presence of various nutrients, all boundary reactions are considered reversible, contributing two parameters to the parameter space.

As an example, we consider the toy model described in the Kelk paper [21] (Figure 1), consisting of 27 reactions, of which, two are boundary, nine are reversible, and EX_Y is the objective reaction; there are 37 parameters in our GSA. The parameter space is sampled with a Sobol low-discrepancy sequence, which is designed for uniform coverage of multidimensional spaces with quasi-random points. With 20 K points, we obtain a stable estimation of the PRCC coefficients (Table 1). As expected, the upper boundaries for reactions R5, R12, and R22 are among the most sensitive parameters, and the upper bounds for reactions R8 and R11 control the reaction module between R5 and R12. The presence of the reversible loop R19-R20-R21-R14 renders reactions R15 and R18 less important for the EX_Y flux. As expected from the model structure, EX_Y flux appears to be sensitive to no one of the lower bound parameters.

### 3.2. FBA-PRCC Can Identify Controlling Steps in the Flux-Based Network

**Figure 2:**
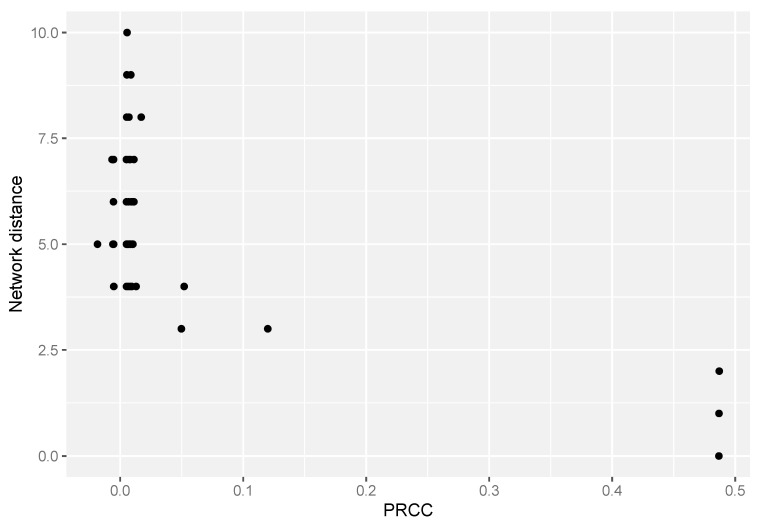
Network distance between vs. PRCC value. For each boundary value network, distance is calculated as a number of reaction steps between the reaction controlled by the boundary value and the objective reaction EX_lys__L_e_u. To avoid influence of hub molecules on the network distances, standard currency metabolites, such as water, ATP, etc., were excluded from network before distance calculations.

For a more biologically relevant example, we calculated the sensitivity of the Lysine production pathway in *E. coli* str. K-12 substr. W3110 (BiGG iEC1372_W3110). Using lower bounds for reversible and boundary reactions and upper bounds for all reactions in the model, we obtained a total of 3730 parameters. The objective coefficient was set to one for the lysine exchange reaction EX_lys__L_e_u. We simulated over 262 K points to obtain reliable values for the PRCC coefficients. Out of 3730 parameters, 55 were significant at the 1% threshold, with 19 lower and 36 upper bounds (Supplementary Table S1). The PRCC plot against the network distance from the objective reactions in Figure 2 shows that the highest sensitivity coefficients correspond to the last three steps of lysine production, including the exchange reaction, transport to extracellular compartment and transport to periplasmic compartment.

**Figure 3:**
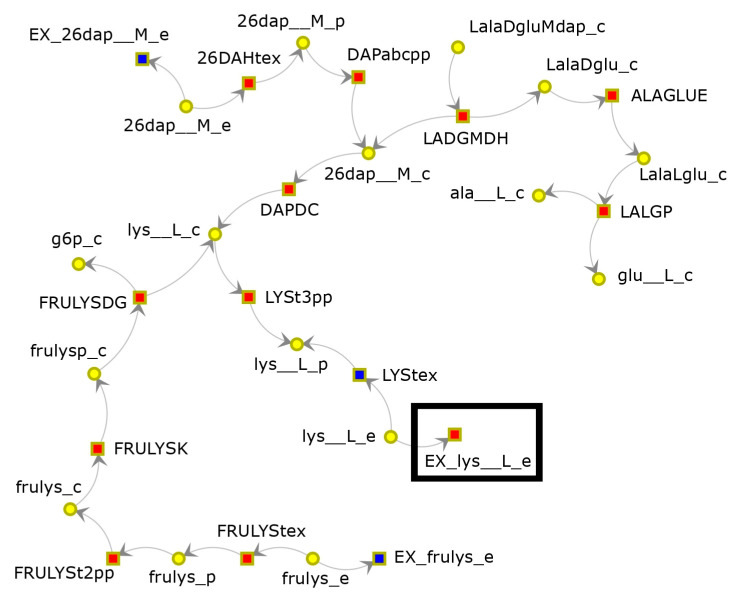
Network of the highly significant (*p*-value > 1%) reactions with positive PRCC values. In this network, squares and circles represent the reactions and metabolites, respectively. The red squares correspond to the reactions with sensitive upper-bound parameters, while the blue squares correspond to the reactions with sensitive lower-bound parameters.

Figure 3 shows that the majority of reactions with positive PRCC values form the backbone of the lysine biosynthesis pathway. Experimental analysis of lysine biosynthesis in *E. coli* has shown that overexpression of diaminopimelate decarboxylase (lysA) and aspartate kinase (lysC) increased lysine titers by 78.1% and 123.6%, respectively [36]. In our model, this corresponds to reactions DAPDC and ASPK, respectively. The PRCC values for the upper boundary of the irreversible DAPDC reaction are 0.12 (*p*-value < 1 × 10^−16^), and for the reversible ASPK reaction, the PRCC coefficients for its upper and lower bounds are 0.0037 (*p*-value 5.9%) and −0.004 (*p*-value 3.9%), respectively. Although FBA-PRCC identifies the reactions important for lysine production and their contribution, the order is different from the experimental data. For instance, DAPDC has a higher PRCC value but a lower increase in lysine production. However, it is important to note that lysine biosynthesis is highly regulated in the cell, as mentioned in [37] and in [36], and accurately describing the regulatory relationships in FBA models is challenging.

The majority of the 15 parameters that are negatively correlated with lysine production correspond to redox balance by decreasing H^+^ production or by shifting NAD/NADH balance.

### 3.3. FBA-PRCC Is Computationally Efficient

Unlike the recently published Sobol variance-based sensitivity analysis for conducting GSA in constraint-based models [23], the FBA-PRCC approach does not require development of special low-level software. All its steps were implemented in Python and R using standard Cobrapy software [24] for FBA calculations and R ‘sensitivity’ package [33]. Calculations of the PRCC values for each flux are independent, so we were using OIST HPC computation clusters to run all these calculations in parallel. Sobol points generation, application to the reaction boundaries, and optimization of objectives were performed in chunks of 8192 per job. Calculations of the PRCC sensitivity coefficients were performed on 262,144 Sobol points in chunks of 10 features per job. The average execution time is 30 min per job for the Sobol point calculations and 7 h per job for the PRCC calculation.

## 4. Discussion

In this work, we have presented a new, fast, and parallelizable framework for estimating the sensitivity coefficients of reaction boundaries in constraint-based models. We demonstrated the performance of our framework using a 27-reaction toy model and the whole-genome metabolic reconstruction of E. coli metabolism. This is the first time that sensitivity coefficients have been calculated for all boundary values in the whole-genome model. Previous analyses, such as those published in [23], have been focused on only a small subset of exchange reactions.

One area where our approach could be particularly useful is in identifying new antibacterial drug targets. We can do this by identifying reaction boundaries that negatively correlate with biomass production and then finding inhibitors for these reactions. Additionally, we can model combinational therapy by analyzing the FBA-PRCC of perturbed models where certain reactions are inhibited or blocked, similar to our analysis in our previous work, Lebedeva et al. [25].

In the future, we plan to expand the application of our approach by exploring its potential in engineering chimeric bacterial cells or microbial communities. To achieve this, we plan to combine our FBA-PRCC method with our metagenomic analysis platform, ASAR [38]. By doing this, we hope to uncover new insights into microbial metabolism and how it can be manipulated for various applications.

Overall, we believe that our approach has the potential to be a powerful tool for both fundamental research and practical applications in biotechnology and medicine.

## Figures and Tables

**Table 1:** PRCC sensitivity coefficients for the toy model, *p*-value calculated according to Marino [14], suffixes ‘u’ and ‘l’ correspond to the upper and the lower bound of the flux, respectively.

Name	Value	*p*-Value	Name	Value	*p*-Value
R1u	0.0472	0	R4u	−0.00183	0.564
R5u	0.388	0	R13u	0.00167	0.597
R8u	0.0398	0	R25u	−0.00162	0.61
R12u	0.0309	0	R21u	0.00157	0.62
R22u	0.0443	0	R4l	0.00114	0.718
R26u	0.0521	0	R10u	0.00101	0.749
EX_Xu	0.83	0	R23l	−0.000947	0.765
R11u	0.0257	4.44 × 10^−16^	R21l	0.000698	0.825
R9u	0.0195	7.72 × 10^−10^	R2l	0.000566	0.858
R2u	0.00868	0.00607	R14l	−0.000326	0.918
R6u	0.00623	0.049	R19u	0.000321	0.919
R15u	0.00523	0.0985	EX_Xl	0.000298	0.925
R7u	0.00518	0.102	R14u	−0.000279	0.93
R18u	0.00488	0.123	R3l	−0.000196	0.951
R3u	0.00482	0.127	R20u	−0.000193	0.951
R16u	0.00317	0.316	R20l	−0.000162	0.959
R24u	0.00285	0.368	R26l	5.99 × 10^−6^	0.998
R17u	0.00269	0.395	R19l	1.72 × 10^−6^	1
R23u	0.00211	0.505			

## Data Availability

The source code and data are available at GitHub: https://github.com/lptolik/FBA-PRCC_paper.

## Supplementary Materials

The following supporting information can be downloaded at: https://www.mdpi.com/article/10.3390/biom13030500/s1, Text S1: PRCC calculation results for lysine production; Model S1: toy model in the SBML format; Table S1: 55 parameters significant at the 1% level.
