# Supplementary material for: FBA-PRCC. Partial Rank Correlation Coefficient (PRCC) Global Sensitivity Analysis (GSA) in Application to Constraint-Based Models
Source: Biomolecules. 2023 Mar 9;13(3):500. doi: 10.3390/biom13030500 (PMC10046323; doi:10.3390/biom13030500)
Supplement: Supplementary file 1 [file biomolecules-13-00500-s001.zip › FBA_PRCC.pdf]

FBA Sensitivity Analysis

A. Sorokin

05.09.2022

1 Read data

1.1 Read PRCC results

Find all CSV data in /Volumes/bucket/GoryaninU/FBA_sensitivity and process them:

```
fl<-dir(bdir,pattern='sobol_data_len11.*.csv')
nums<-as.numeric(sub('sobol_data_len11_40_([0-9]+).csv','\\1',fl))
```

There are datasets for 8192, 8.192×10^4, 9.8304×10^4, 1.14688×10^5, 1.31072×10^5, 1.47456×10^5, 1.6384×10^4, 2.62144×10^5, 3.2768×10^4, 6.5536×10^4 random points.

```
fl<-fl[order(nums)]
ranks<-matrix(NA,ncol = length(unique(nums)),nrow = 3730)
i<-1
 for(f in fl){
 df<-read.csv(file.path(bdir,f),sep='\t')
 df<-df[order(df$varnum),]
 cat(f,dim(df),'\n')
 ranks[,i]<-rank(-abs(df$original),ties.method= "first")
 i<-i+1
 }
```

```
## sobol_data_len11_40_1.csv 3730 6
## sobol_data_len11_40_2.csv 3730 6
## sobol_data_len11_40_4.csv 3730 6
## sobol_data_len11_40_8.csv 3730 6
## sobol_data_len11_40_10.csv 3730 6
## sobol_data_len11_40_12.csv 3730 6
## sobol_data_len11_40_14.csv 3730 6
## sobol_data_len11_40_16.csv 3730 6
## sobol_data_len11_40_18.csv 3730 6
## sobol_data_len11_40_32.csv 3730 6
```

```
r1<-ODEsensitivity::tdcc(t(ranks[,(length(nums)-1:0)]),pearson = TRUE,plot = TRUE)
```

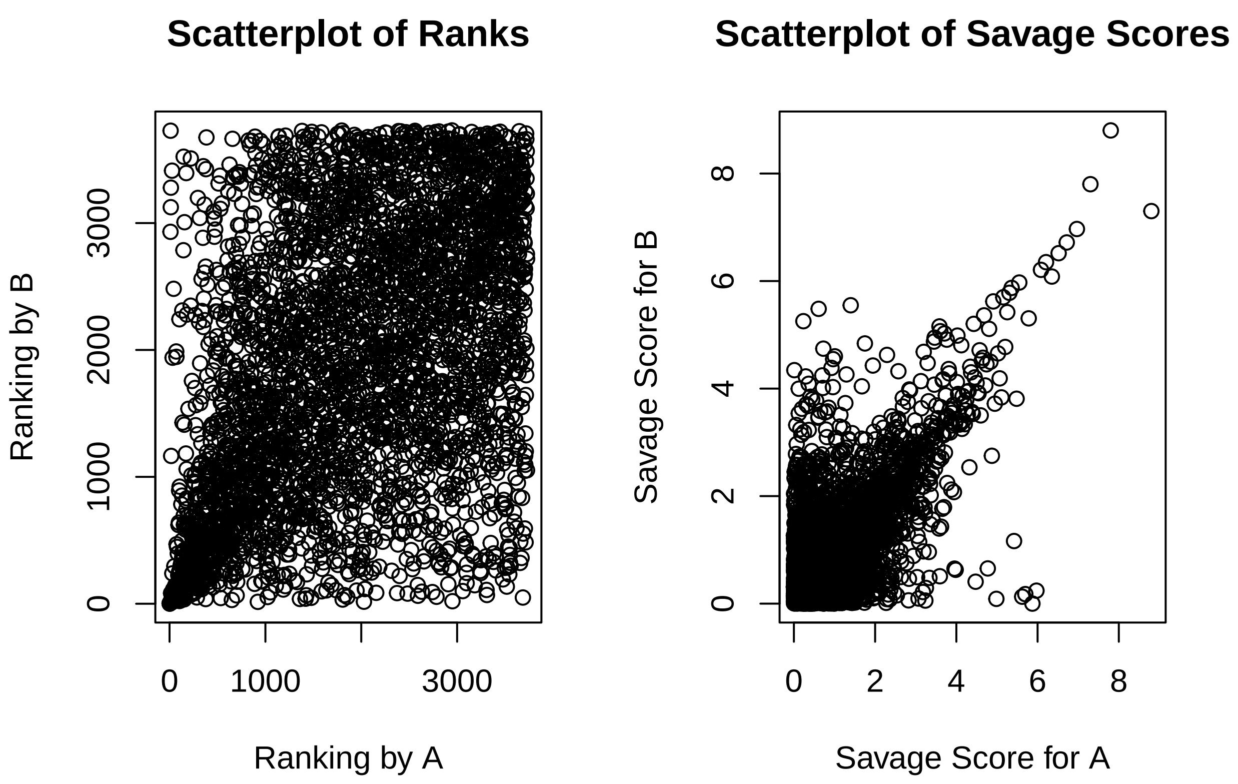


```
r1
```

```
##        kendall       pearson
## -1.491817e+04  6.534186e-01
```

1.2 Lysine analysis

```
df001<-df[df$pval<0.01,]
row.names(df001)<-df001$varname
pander(df001[order(df001$original),c("original","gdist","pval","ID")],
       justify = c('left', 'center', 'center', 'center', 'left'))
```

	original	gdist	pval	ID
HACD2i_u	-0.0184	5	0	HACD2i
EX_lcts_e_u	-0.00661	7	0.000778	EX_lcts_e
NOVBCNtpp_u	-0.00624	NA	0.0015	NOVBCNtpp
EX_fe3hox_e_l	-0.00595	7	0.00251	EX_fe3hox_e
HACD1_l	-0.00591	5	0.00264	HACD1
4PCPpp_u	-0.00582	5	0.0031	4PCPpp
NO2t2rpp_l	-0.00559	7	0.00452	NO2t2rpp
FUSAtex_l	-0.00547	NA	0.00545	FUSAtex
ACALDtex_l	-0.00545	6	0.00559	ACALDtex
CTBTt2rpp_l	-0.00541	NA	0.00597	CTBTt2rpp
NO3tex_u	-0.00539	7	0.00616	NO3tex
ARGDCpp_u	-0.00538	5	0.0062	ARGDCpp
EX_pyr_e_l	-0.00535	7	0.00651	EX_pyr_e
CPGNabcpp_u	-0.00525	5	0.00761	CPGNabcpp
CFAS180E_u	-0.00517	4	0.00854	CFAS180E
MEOHtex_l	0.00512	7	0.00927	MEOHtex

	original	gdist	pval	ID
PRMICI_l	0.00513	5	0.00905	PRMICI
ACACtex_l	0.00517	6	0.00863	ACACtex
MTHFC_l	0.00519	4	0.00835	MTHFC
ACONTa_u	0.0052	5	0.00817	ACONTa
HCYSMT2_u	0.00524	4	0.00772	HCYSMT2
EX_gam6p_e_u	0.00531	7	0.00698	EX_gam6p_e
EX_dad_2_e_l	0.00538	8	0.00626	EX_dad_2_e
EX_glcr_e_l	0.00551	9	0.00513	EX_glcr_e
EX_rmn_e_u	0.0057	10	0.00377	EX_rmn_e
LPLIPAL2A161_u	0.00587	5	0.00285	LPLIPAL2A161
LEUt2rpp_l	0.00601	4	0.00223	LEUt2rpp
ALAGLUE_u	0.00624	5	0.00151	ALAGLUE
LADGMDH_u	0.00681	4	0.000542	LADGMDH
PSCLYSt2pp_u	0.00692	6	0.000439	PSCLYSt2pp
EX_psclys_e_l	0.00718	8	0.00026	EX_psclys_e
MDH2_u	0.0072	5	0.000251	MDH2
PSCLYStex_u	0.00745	7	0.000153	PSCLYStex
LALGP_u	0.00761	4	0.000108	LALGP
EX_akg_e_l	0.00809	7	3.93e-05	EX_akg_e
GRTT_u	0.0084	5	1.95e-05	GRTT
EX_3ump_e_u	0.00874	9	8.82e-06	EX_3ump_e
ADNCYC_u	0.00915	NA	3.26e-06	ADNCYC
FRULYStex_u	0.00917	6	3.14e-06	FRULYStex
DAPabcpp_u	0.00945	4	1.54e-06	DAPabcpp
AGM3PH_u	0.00958	6	1.12e-06	AGM3PH
FEOXAMabcpp_u	0.00985	5	5.51e-07	FEOXAMabcpp
SELNPS_u	0.0101	6	3.12e-07	SELNPS
26DAHtex_u	0.0102	5	2.18e-07	26DAHtex
FRULYSt2pp_u	0.0104	5	1.23e-07	FRULYSt2pp
**EX_26dap__M_e_l**	0.0112	6	1.27e-08	EX_26dap__M_e
EX_frulys_e_l	0.0112	7	1.26e-08	EX_frulys_e
MOHMT_u	0.0129	4	4.63e-11	MOHMT
CBMD_u	0.0172	8	0	CBMD
FRULYSDG_u	0.0498	3	0	FRULYSDG
FRULYSK_u	0.052	4	0	FRULYSK
DAPDC_u	0.12	3	0	DAPDC
**EX_lys__L_e_u**	0.487	0	0	EX_lys__L_e
LYStex_l	0.487	1	0	LYStex
LYSt3pp_u	0.487	2	0	LYSt3pp

```
pander(df001[order(df001$original),c("original","gdist","Name")],
      justify = c('left', 'center', 'center', 'left'))
```

	original	gdist	Name
HACD2i_u	-0.0184	5	3-hydroxyacyl-CoA dehydrogenase (3-oxohexanoyl-CoA)
EX_lcts_e_u	-0.00661	7	Lactose exchange
NOVBCNtpp_u	-0.00624	NA	Novobiocin transport via TolC system
EX_fe3hox_e_l	-0.00595	7	Fe(III)hydroxamate exchange
HACD1_l	-0.00591	5	3-hydroxyacyl-CoA dehydrogenase (acetoacetyl-CoA)
4PCPpp_u	-0.00582	5	Tetrapeptide L,D-carboxypeptidase (periplasm)
NO2t2rpp_l	-0.00559	7	Nitrite transport in via proton symport, reversible (periplasm)

	original	gdist	Name
FUSAtex_l	-0.00547	NA	Fusidic acid transport via diffusion (extracellular to periplasm)
ACALDtex_l	-0.00545	6	Acetaldehyde transport via diffusion (extracellular to periplasm)
CTBTt2rpp_l	-0.00541	NA	Cronobetaine outward transport (H+ antiport)
NO3tex_u	-0.00539	7	Nitrate transport via diffusion (extracellular to periplasm)
ARGDCpp_u	-0.00538	5	Arginine decarboxylase
EX_pyr_e_l	-0.00535	7	Pyruvate exchange
CPGNabcpp_u	-0.00525	5	Coprogen transport via ABC system (periplasm)
CFAS180E_u	-0.00517	4	Cyclopropane fatty acid synthase (Phosphatidylethanolamine, n-C18:0)
MEOHtex_l	0.00512	7	Methanol transport via diffusion (extracellular to periplasm)
PRMICI_l	0.00513	5	1-(5-phosphoribosyl)-5-[(5-phosphoribosylamino)methylideneamino)imidazole-4-carboxamide isomerase
ACACtex_l	0.00517	6	Acetoacetate transport via diffusion (extracellular to periplasm)
MTHFC_l	0.00519	4	Methenyltetrahydrofolate cyclohydrolase
ACONTa_u	0.0052	5	Aconitase (half-reaction A, Citrate hydro-lyase)
HCYSMT2_u	0.00524	4	Homocysteine Methyltransferase
EX_gam6p_e_u	0.00531	7	D-Glucosamine 6-phosphate exchange
EX_dad_2_e_l	0.00538	8	Deoxyadenosine exchange
EX_glcr_e_l	0.00551	9	D-Glucarate exchange
EX_rmn_e_u	0.0057	10	L-Rhamnose exchange
LPLIPAL2A161_u	0.00587	5	Lysophospholipase L2 (2-acylglycerophosphotidate, n-C16:1)
LEUt2rpp_l	0.00601	4	L-leucine reversible transport via proton symport (periplasm)
ALAGLUE_u	0.00624	5	L-alanyl-gamma-glutamate epimerase
LADGMDH_u	0.00681	4	L-alanyl-gamma-D-glutamyl-meso-diaminopimelate hydrolase
PSCLYSt2pp_u	0.00692	6	Psicoselysine transport via proton symport (periplasm)
EX_psclys_e_l	0.00718	8	Psicoselysine exchange
MDH2_u	0.0072	5	Malate dehydrogenase (ubiquinone 8 as acceptor)
PSCLYStex_u	0.00745	7	Psicoselysine transporter via diffusion (extracellular)
LALGP_u	0.00761	4	L-alanyl-gamma-L-glutamate peptidase
EX_akg_e_l	0.00809	7	2-Oxoglutarate exchange
GRTT_u	0.0084	5	Geranyltranstransferase
EX_3ump_e_u	0.00874	9	3'-UMP exchange
ADNCYC_u	0.00915	NA	Adenylate cyclase
FRULYStex_u	0.00917	6	Fructoselysine transporter via diffusion (extracellular)
DAPabcpp_u	0.00945	4	M-diaminopimelic acid ABC transport (periplasm)
AGM3PH_u	0.00958	6	N-Acetyl-D-glucosamine(anhydrous)N-Acetylmuramyl-tripeptide beta -1,4-N-acetylglucosaminidase
FEOXAMabcpp_u	0.00985	5	Ferroxamine transport via ABC system (periplasm)
SELNPS_u	0.0101	6	Selenophosphate synthase
26DAHtex_u	0.0102	5	Meso-2,6-Diaminoheptanedioate transport via diffusion (extracellular to periplasm)
FRULYSt2pp_u	0.0104	5	Fructoselysine transport via proton symport (periplasm)
**EX_26dap__M_e_l**	0.0112	6	Meso-2,6-Diaminoheptanedioate exchange
EX_frulys_e_l	0.0112	7	Fructoselysine exchange
MOHMT_u	0.0129	4	3-methyl-2-oxobutanoate hydroxymethyltransferase
CBMD_u	0.0172	8	Carbamate deaminase
FRULYSDG_u	0.0498	3	Fructoselysine phosphate deglycase
FRULYSK_u	0.052	4	Fructoselysine Kinase
DAPDC_u	0.12	3	Diaminopimelate decarboxylase
**EX_lys__L_e_u**	0.487	0	L-Lysine exchange
LYStex_l	0.487	1	L-lysine transport via diffusion (extracellular to periplasm)

	original	gdist	Name
LYSt3pp_u	0.487	2	L-lysine transport out via proton antiport (cytoplasm to periplasm)

```
pander(df001[order(df001$original),c("original","gdist","Equation")],
      justify = c('left', 'center', 'center', 'left'))
```

	original	gdist	Equation
HACD2i_u	-0.0184	5	3hhcoa_c + nad_c –> 3ohcoa_c + h_c + nadh_c
EX_lcts_e_u	-0.00661	7	lcts_e –>
NOVBCNtpp_u	-0.00624	NA	h_p + novbcn_p –> h_c + novbcn_e
EX_fe3hox_e_l	-0.00595	7	fe3hox_e –>
HACD1_l	-0.00591	5	aacoa_c + h_c + nadh_c <=> 3hbcoa_c + nad_c
4PCPpp_u	-0.00582	5	LalaDgluMdapDala_p + h2o_p –> LalaDgluMdap_p + ala__D_p
NO2t2rpp_l	-0.00559	7	h_p + no2_p <=> h_c + no2_c
FUSAtex_l	-0.00547	NA	fusa_e <=> fusa_p
ACALDtex_l	-0.00545	6	acald_e <=> acald_p
CTBTt2rpp_l	-0.00541	NA	ctbt_p + h_p <=> ctbt_c + h_c
NO3tex_u	-0.00539	7	no3_e <=> no3_p
ARGDCpp_u	-0.00538	5	arg__L_p + h_p –> agm_p + co2_p
EX_pyr_e_l	-0.00535	7	pyr_e –>
CPGNabcpp_u	-0.00525	5	atp_c + cpgn_p + h2o_c –> adp_c + cpgn_c + h_c + pi_c
CFAS180E_u	-0.00517	4	2.0 amet_c + pe181_c –> 2.0 ahcys_c + cpe180_c + 2.0 h_c
MEOHtex_l	0.00512	7	meoh_e <=> meoh_p
PRMICI_l	0.00513	5	prfp_c <=> prlp_c
ACACtex_l	0.00517	6	acac_e <=> acac_p
MTHFC_l	0.00519	4	h2o_c + methf_c <=> 10fthf_c + h_c
ACONTa_u	0.0052	5	cit_c <=> acon_C_c + h2o_c
HCYSMT2_u	0.00524	4	hcys__L_c + mmet_c –> h_c + 2.0 met__L_c
EX_gam6p_e_u	0.00531	7	gam6p_e –>
EX_dad_2_e_l	0.00538	8	dad_2_e –>
EX_glcr_e_l	0.00551	9	glcr_e –>
EX_rmn_e_u	0.0057	10	rmn_e –>
LPLIPAL2A161_u	0.00587	5	2hdec9eg3p_c + h2o_c –> glyc3p_c + 2.0 h_c + hdcea_c
LEUt2rpp_l	0.00601	4	h_p + leu__L_p <=> h_c + leu__L_c
ALAGLUE_u	0.00624	5	LalaDglu_c <=> LalaLglu_c
LADGMDH_u	0.00681	4	LalaDgluMdap_c + h2o_c –> 26dap__M_c + LalaDglu_c
PSCLYSt2pp_u	0.00692	6	h_p + psclys_p –> h_c + psclys_c
EX_psclys_e_l	0.00718	8	psclys_e –>
MDH2_u	0.0072	5	mal__L_c + q8_c –> oaa_c + q8h2_c
PSCLYStex_u	0.00745	7	psclys_e <=> psclys_p
LALGP_u	0.00761	4	LalaLglu_c + h2o_c –> ala__L_c + glu__L_c
EX_akg_e_l	0.00809	7	akg_e –>
GRTT_u	0.0084	5	grdp_c + ipdp_c –> frdp_c + ppi_c

	original	gdist	Equation
EX_3ump_e_u	0.00874	9	3ump_e ->
ADNCYC_u	0.00915	NA	atp_c -> camp_c + ppi_c
FRULYStex_u	0.00917	6	frulys_e <=> frulys_p
DAPabcpp_u	0.00945	4	26dap__M_p + atp_c + h2o_c -> 26dap__M_c + adp_c + h_c + pi_c
AGM3PH_u	0.00958	6	anhgm3p_c + h2o_c -> acgam_c + anhm3p_c
FEOXAMabcpp_u	0.00985	5	atp_c + feoxam_p + h2o_c -> adp_c + feoxam_c + h_c + pi_c
SELNPS_u	0.0101	6	atp_c + h2o_c + seln_c -> amp_c + pi_c + selnp_c
26DAHtex_u	0.0102	5	26dap__M_e <=> 26dap__M_p
FRULYSt2pp_u	0.0104	5	frulys_p + h_p -> frulys_c + h_c
**EX_26dap__M_e_l**	0.0112	6	26dap__M_e ->
EX_frulys_e_l	0.0112	7	frulys_e ->
MOHMT_u	0.0129	4	3mob_c + h2o_c + mlthf_c -> 2dhp_c + thf_c
CBMD_u	0.0172	8	cbm_c + 2.0 h_c -> co2_c + nh4_c
FRULYSDG_u	0.0498	3	frulysp_c + h2o_c <=> g6p_c + lys__L_c
FRULYSK_u	0.052	4	atp_c + frulys_c -> adp_c + frulysp_c + h_c
DAPDC_u	0.12	3	26dap__M_c + h_c -> co2_c + lys__L_c
**EX_lys__L_e_u**	0.487	0	lys__L_e ->
LYStex_l	0.487	1	lys__L_e <=> lys__L_p
LYSt3pp_u	0.487	2	h_p + lys__L_c -> h_c + lys__L_p

```
qplot(original,gdist,data=df001)+xlab('PRCC')+ylab('Network distance')
```

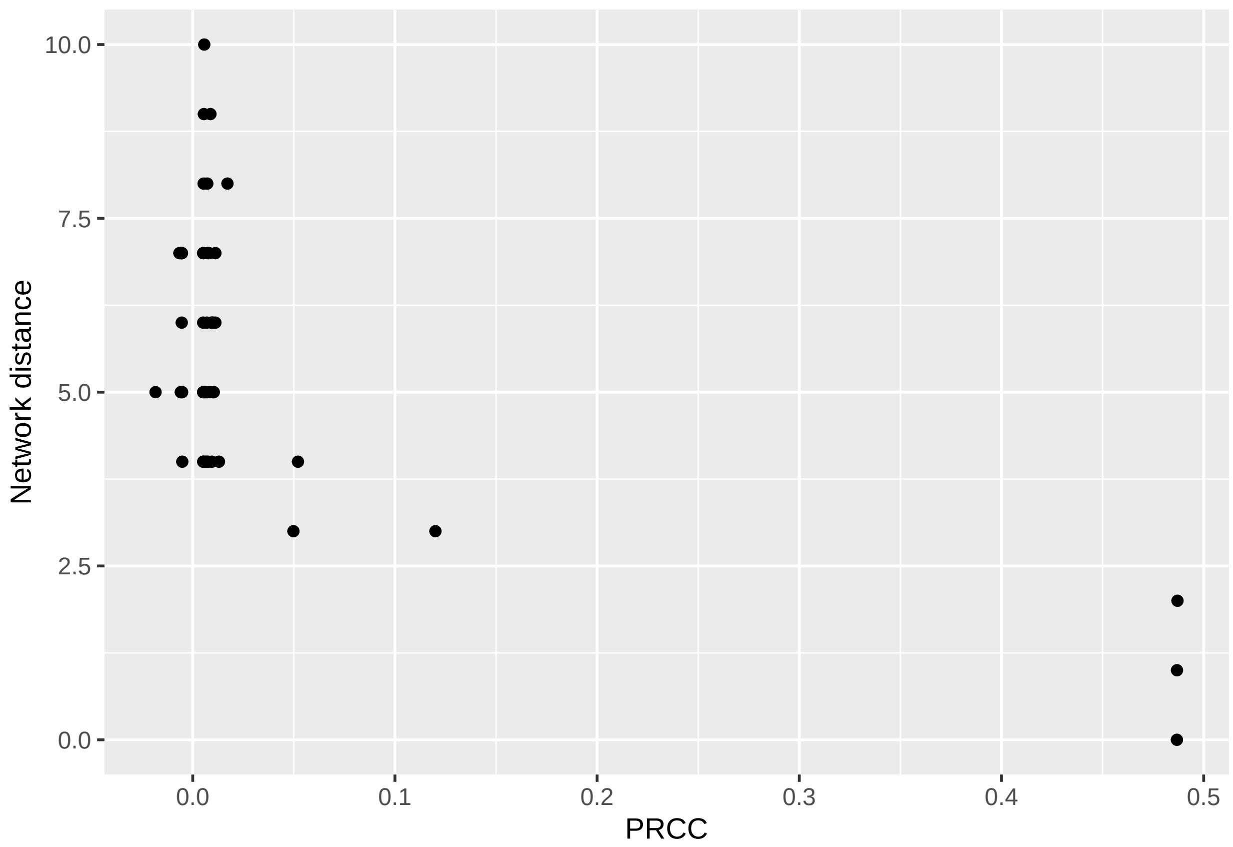


```
df005<-df[df$pval<0.05,]
row.names(df005)<-df005$varname
pander(df005[order(df005$original),c("original","gdist","pval","ID")],
       justify = c('left', 'center', 'center', 'center', 'left'))
```

	original	gdist	pval	ID
HACD2i_u	-0.0184	5	0	HACD2i
EX_lcts_e_u	-0.00661	7	0.000778	EX_lcts_e
NOVBCNtpp_u	-0.00624	NA	0.0015	NOVBCNtpp
EX_fe3hox_e_l	-0.00595	7	0.00251	EX_fe3hox_e
HACD1_l	-0.00591	5	0.00264	HACD1
4PCPpp_u	-0.00582	5	0.0031	4PCPpp
NO2t2rpp_l	-0.00559	7	0.00452	NO2t2rpp
FUSAtex_l	-0.00547	NA	0.00545	FUSAtex
ACALDtex_l	-0.00545	6	0.00559	ACALDtex
CTBTt2rpp_l	-0.00541	NA	0.00597	CTBTt2rpp
NO3tex_u	-0.00539	7	0.00616	NO3tex
ARGDCpp_u	-0.00538	5	0.0062	ARGDCpp
EX_pyr_e_l	-0.00535	7	0.00651	EX_pyr_e
CPGNabcpp_u	-0.00525	5	0.00761	CPGNabcpp
CFAS180E_u	-0.00517	4	0.00854	CFAS180E
A5PISO_l	-0.00505	6	0.0103	A5PISO
CU2tex_u	-0.00504	5	0.0105	CU2tex
GLUNpp_u	-0.00497	5	0.0116	GLUNpp
MDRPD_u	-0.00489	6	0.013	MDRPD
PROt2rpp_l	-0.00487	4	0.0133	PROt2rpp

	original	gdist	pval	ID
EX_3hpp_e_u	-0.00486	13	0.0134	EX_3hpp_e
**EX_hom__L_e_l**	-0.00482	7	0.0142	EX_hom__L_e
EX_damp_e_l	-0.00482	9	0.0143	EX_damp_e
FACOAE161_u	-0.00481	5	0.0145	FACOAE161
AACPS9_u	-0.00479	6	0.0149	AACPS9
HPPPNtex_u	-0.00478	9	0.0151	HPPPNtex
GLTPD_l	-0.00469	6	0.0171	GLTPD
HEPTA1_u	-0.00464	7	0.0184	HEPTA1
3hoxpactex_l	-0.00459	NA	0.0197	3hoxpactex
UDPGtex_l	-0.00458	7	0.0198	UDPGtex
MCPST_u	-0.00455	5	0.0207	MCPST
GLYC2Ptex_u	-0.00454	6	0.0211	GLYC2Ptex
NTD9_u	-0.00453	5	0.0214	NTD9
PGI_l	-0.00453	4	0.0214	PGI
CBLAT_l	-0.00452	4	0.0217	CBLAT
UM4PCP_u	-0.00445	5	0.0237	UM4PCP
HCINNMt2rpp_u	-0.00442	8	0.0248	HCINNMt2rpp
GLUABUTt7pp_u	-0.00439	4	0.0256	GLUABUTt7pp
M1PD_u	-0.00434	5	0.0275	M1PD
PLIPA2A120pp_u	-0.00433	6	0.0277	PLIPA2A120pp
ACLS_u	-0.00428	5	0.0294	ACLS
EX_cynt_e_l	-0.00421	8	0.0324	EX_cynt_e
2AGPG161tipp_u	-0.0042	5	0.0328	2AGPG161tipp
MALDDH_u	-0.00419	5	0.0333	MALDDH
GLCURt2rpp_u	-0.00416	9	0.0347	GLCURt2rpp
AMAOTr_l	-0.00411	4	0.0365	AMAOTr
ASPK_l	-0.00404	4	0.0399	ASPK
SERtex_l	-0.00404	5	0.0401	SERtex
MOX_u	-0.00402	5	0.0412	MOX
ASO3tex_l	-0.004	9	0.0418	ASO3tex
FALDH2_l	-0.00396	6	0.0444	FALDH2
GLCNt2rpp_u	-0.00392	6	0.0461	GLCNt2rpp
ANS_u	-0.00392	4	0.0462	ANS
NAtex_l	-0.00392	NA	0.0464	NA
FCLPA_l	-0.00388	5	0.0485	FCLPA
ME1_u	-0.00388	5	0.0488	ME1
PRPPS_l	-0.00386	5	0.0499	PRPPS
UREAtpp_l	0.00386	5	0.0497	UREAtpp
EX_r5p_e_u	0.00388	9	0.0488	EX_r5p_e
ALAALAr_u	0.00391	5	0.0466	ALAALAr
FEOXAMUtex_u	0.00393	6	0.0459	FEOXAMUtex
PUNP6_l	0.00402	6	0.0412	PUNP6
NDPK3_l	0.00404	4	0.0402	NDPK3
CLPNS141pp_l	0.00407	5	0.0385	CLPNS141pp
EX_23ccmp_e_l	0.00408	10	0.0382	EX_23ccmp_e
NDPK7_l	0.00411	4	0.0365	NDPK7
EX_glyald_e_l	0.00414	9	0.0352	EX_glyald_e
NO3R2bpp_u	0.00415	7	0.0348	NO3R2bpp
F6PA_l	0.00421	5	0.0322	F6PA
SPMDabcpp_u	0.00424	4	0.031	SPMDabcpp
FRULYStex_l	0.00426	6	0.0302	FRULYStex
EX_slnt_e_l	0.00431	10	0.0285	EX_slnt_e

	original	gdist	pval	ID
GLYC2Ptex_l	0.00431	6	0.0284	GLYC2Ptex
EX_acgam1p_e_u	0.00431	8	0.0284	EX_acgam1p_e
DINStex_l	0.00432	8	0.028	DINStex
SUCBZL_u	0.00435	6	0.0271	SUCBZL
EX_ocdca_e_l	0.00443	8	0.0244	EX_ocdca_e
EX_cl_e_l	0.00446	6	0.0233	EX_cl_e
EX_hg2_e_l	0.00449	NA	0.0225	EX_hg2_e
PTAr_u	0.00451	4	0.0218	PTAr
DALAtex_l	0.0046	5	0.0193	DALAtex
EX_g3pe_e_l	0.00462	7	0.0189	EX_g3pe_e
G3PD7_u	0.00463	4	0.0186	G3PD7
EX_g3pc_e_l	0.0047	8	0.0168	EX_g3pc_e
APRAUR_u	0.00481	6	0.0145	APRAUR
ECOAH8_l	0.00483	6	0.014	ECOAH8
PLIPA2A180pp_u	0.00489	6	0.0129	PLIPA2A180pp
GALAMtex_u	0.00491	NA	0.0125	GALAMtex
SUCBZS_u	0.005	5	0.0111	SUCBZS
MEOHtex_l	0.00512	7	0.00927	MEOHtex
PRMICI_l	0.00513	5	0.00905	PRMICI
ACACtex_l	0.00517	6	0.00863	ACACtex
MTHFC_l	0.00519	4	0.00835	MTHFC
ACONTa_u	0.0052	5	0.00817	ACONTa
HCYSMT2_u	0.00524	4	0.00772	HCYSMT2
EX_gam6p_e_u	0.00531	7	0.00698	EX_gam6p_e
EX_dad_2_e_l	0.00538	8	0.00626	EX_dad_2_e
EX_glcr_e_l	0.00551	9	0.00513	EX_glcr_e
EX_rmn_e_u	0.0057	10	0.00377	EX_rmn_e
LPLIPAL2A161_u	0.00587	5	0.00285	LPLIPAL2A161
LEUt2rpp_l	0.00601	4	0.00223	LEUt2rpp
ALAGLUE_u	0.00624	5	0.00151	ALAGLUE
LADGMDH_u	0.00681	4	0.000542	LADGMDH
PSCLYSt2pp_u	0.00692	6	0.000439	PSCLYSt2pp
EX_psclys_e_l	0.00718	8	0.00026	EX_psclys_e
MDH2_u	0.0072	5	0.000251	MDH2
PSCLYStex_u	0.00745	7	0.000153	PSCLYStex
LALGP_u	0.00761	4	0.000108	LALGP
EX_akg_e_l	0.00809	7	3.93e-05	EX_akg_e
GRTT_u	0.0084	5	1.95e-05	GRTT
EX_3ump_e_u	0.00874	9	8.82e-06	EX_3ump_e
ADNCYC_u	0.00915	NA	3.26e-06	ADNCYC
FRULYStex_u	0.00917	6	3.14e-06	FRULYStex
DAPabcpp_u	0.00945	4	1.54e-06	DAPabcpp
AGM3PH_u	0.00958	6	1.12e-06	AGM3PH
FEOXAMabcpp_u	0.00985	5	5.51e-07	FEOXAMabcpp
SELNPS_u	0.0101	6	3.12e-07	SELNPS
26DAHtex_u	0.0102	5	2.18e-07	26DAHtex
FRULYSt2pp_u	0.0104	5	1.23e-07	FRULYSt2pp
**EX_26dap__M_e_l**	0.0112	6	1.27e-08	EX_26dap__M_e
EX_frulys_e_l	0.0112	7	1.26e-08	EX_frulys_e
MOHMT_u	0.0129	4	4.63e-11	MOHMT
CBMD_u	0.0172	8	0	CBMD
FRULYSDG_u	0.0498	3	0	FRULYSDG

	original	gdist	pval	ID
FRULYSK_u	0.052	4	0	FRULYSK
DAPDC_u	0.12	3	0	DAPDC
**EX_lys__L_e_u**	0.487	0	0	EX_lys__L_e
LYStex_l	0.487	1	0	LYStex
LYSt3pp_u	0.487	2	0	LYSt3pp

```
pander(df005[order(df005$original),c("original","gdist","Name")],
      justify = c('left', 'center', 'center', 'left'))
```

	original	gdist	Name
HACD2i_u	-0.0184	5	3-hydroxyacyl-CoA dehydrogenase (3-oxohexanoyl-CoA)
EX_lcts_e_u	-0.00661	7	Lactose exchange
NOVBCNtpp_u	-0.00624	NA	Novobiocin transport via TolC system
EX_fe3hox_e_l	-0.00595	7	Fe(III)hydroxamate exchange
HACD1_l	-0.00591	5	3-hydroxyacyl-CoA dehydrogenase (acetoacetyl-CoA)
4PCPpp_u	-0.00582	5	Tetrapeptide L,D-carboxypeptidase (periplasm)
NO2t2rpp_l	-0.00559	7	Nitrite transport in via proton symport, reversible (periplasm)
FUSAtex_l	-0.00547	NA	Fusidic acid transport via diffusion (extracellular to periplasm)
ACALDtex_l	-0.00545	6	Acetaldehyde transport via diffusion (extracellular to periplasm)
CTBTt2rpp_l	-0.00541	NA	Cronobetaine outward transport (H+ antiport)
NO3tex_u	-0.00539	7	Nitrate transport via diffusion (extracellular to periplasm)
ARGDCpp_u	-0.00538	5	Arginine decarboxylase
EX_pyr_e_l	-0.00535	7	Pyruvate exchange
CPGNabcpp_u	-0.00525	5	Coprogen transport via ABC system (periplasm)
CFAS180E_u	-0.00517	4	Cyclopropane fatty acid synthase (Phosphatidylethanolamine, n-C18:0)
A5PISO_l	-0.00505	6	Arabinose-5-phosphate isomerase
CU2tex_u	-0.00504	5	Copper (Cu+2) transport via diffusion (extracellular to periplasm)
GLUNpp_u	-0.00497	5	Glutaminase
MDRPD_u	-0.00489	6	5-Methylthio-5-deoxy-D-ribulose 1-phosphate dehydratase
PROt2rpp_l	-0.00487	4	L-proline reversible transport via proton symport (periplasm)
EX_3hpp_e_u	-0.00486	13	3-Hydroxypropanoate exchange
**EX_hom__L_e_u**	-0.00482	7	L-Homoserine exchange
EX_damp_e_l	-0.00482	9	DAMP exchange
FACOAE161_u	-0.00481	5	Fatty-acid-CoA thioesterase (hexadecenoate)
AACPS9_u	-0.00479	6	Acyl-[acyl-carrier-protein] synthetase (n-C8:0)
HPPPNtex_u	-0.00478	9	3-(3-hydroxyphenyl)propionate transport via diffusion (extracellular to periplasm)
GLTPD_l	-0.00469	6	Galactitol-1-phosphate dehydrogenase
HEPTA1_u	-0.00464	7	heptosyltransferase I LPS core synthesis
3hoxpactex_l	-0.00459	NA	3 hydroxyphenylacetic acid transport via diffusion extracellular to periplasm
UDPGtex_l	-0.00458	7	UDPglucose transport via diffusion (extracellular to periplasm)
MCPST_u	-0.00455	5	3-mercaptopyruvate sulfurtransferase
GLYC2Ptex_u	-0.00454	6	Glycerol-2-phosphate transport via diffusion (extracellular to periplasm)
NTD9_u	-0.00453	5	5'-nucleotidase (GMP)
PGI_l	-0.00453	4	Glucose-6-phosphate isomerase
CBLAT_l	-0.00452	4	Cob(I)alamin adenosyltransferase
UM4PCP_u	-0.00445	5	UDP-N-acetylmuramoyl-L-alanyl-D-gamma-glutamyl-meso-2,6-diaminopimelate-D-alanine L,D-carboxypeptidase

	original	gdist	Name
HCINNMt2rpp_u	-0.00442	8	3-hydroxycinnamic acid transport via proton symport, reversible (periplasm)
GLUABUTt7pp_u	-0.00439	4	4-aminobutyrate/glutamate antiport (periplasm)
M1PD_u	-0.00434	5	Mannitol-1-phosphate 5-dehydrogenase
PLIPA2A120pp_u	-0.00433	6	Phospholipase A2 (phosphatidate, n-C12:0) (periplasm)
ACLS_u	-0.00428	5	Acetolactate synthase
EX_cynt_e_l	-0.00421	8	Cyanate exchange
2AGPG161tipp_u	-0.0042	5	2-Acyl-sn-glycero-3-phosphoglycerol (n-C16:1) transporter via facilitated diffusion (periplasm)
MALDDH_u	-0.00419	5	Malate decarboxylating oxidoreductase (decarboxylating)
GLCURt2rpp_u	-0.00416	9	D-glucuronate transport via proton symport, reversible (periplasm)
AMAOTr_l	-0.00411	4	Adenosylmethionine-8-amino-7-oxononanoate transaminase
ASPK_l	-0.00404	4	Aspartate kinase
SERtex_l	-0.00404	5	L-serine transport via diffusion (extracellular to periplasm)
MOX_u	-0.00402	5	Malate oxidase
ASO3tex_l	-0.004	9	Arsenite transport via diffusion (extracellular to periplasm)
FALDH2_l	-0.00396	6	Formaldehyde dehydrogenase
GLCNt2rpp_u	-0.00392	6	D-gluconate transport via proton symport, reversible (periplasm)
ANS_u	-0.00392	4	Anthranilate synthase
NAtex_l	-0.00392	NA	NA
FCLPA_l	-0.00388	5	L-fuculose 1-phosphate aldolase
ME1_u	-0.00388	5	Malic enzyme (NAD)
PRPPS_l	-0.00386	5	Phosphoribosylpyrophosphate synthetase
UREAtpp_l	0.00386	5	Urea transport via facilitate diffusion (periplasm)
EX_r5p_e_u	0.00388	9	Alpha-D-Ribose 5-phosphate exchange
ALAALAr_u	0.00391	5	D-alanine-D-alanine ligase (reversible)
FEOXAMUtex_u	0.00393	6	Ferroxamine (minus Fe3) secretion (to extracellular)
PUNP6_l	0.00402	6	Purine-nucleoside phosphorylase (Deoxyinosine)
NDPK3_l	0.00404	4	Nucleoside-diphosphate kinase (ATP:CDP)
CLPNS141pp_l	0.00407	5	Cardiolipin synthase (periplasmic, n-C14:1)
EX_23ccmp_e_l	0.00408	10	2',3'-Cyclic CMP exchange
NDPK7_l	0.00411	4	Nucleoside-diphosphate kinase (ATP:dCDP)
EX_glyald_e_l	0.00414	9	D-Glyceraldehyde exchange
NO3R2bpp_u	0.00415	7	Nitrate reductase (Menaquinol-8) (periplasm)
F6PA_l	0.00421	5	Fructose 6-phosphate aldolase
SPMDabcpp_u	0.00424	4	Spermidine transport via ABC system (periplasm)
FRULYStex_l	0.00426	6	Fructoselysine transporter via diffusion (extracellular)
EX_slnt_e_l	0.00431	10	Selenite exchange
GLYC2Ptex_l	0.00431	6	Glycerol-2-phosphate transport via diffusion (extracellular to periplasm)
EX_acgam1p_e_u	0.00431	8	N-Acetyl-D-glucosamine 1-phosphate exchange
DINStex_l	0.00432	8	Deoxyinosine transport via diffusion (extracellular to periplasm)
SUCBZL_u	0.00435	6	O-succinylbenzoate-CoA ligase
EX_ocdca_e_l	0.00443	8	Octadecanoate (n-C18:0) exchange
EX_cl_e_l	0.00446	6	Chloride exchange
EX_hg2_e_l	0.00449	NA	Hg2+ exchange
PTAr_u	0.00451	4	Phosphotransacetylase
DALAtex_l	0.0046	5	D-Alanine transport via diffusion (extracellular to periplasm)
EX_g3pe_e_l	0.00462	7	Sn-Glycero-3-phosphoethanolamine exchange
G3PD7_u	0.00463	4	Glycerol-3-phosphate dehydrogenase (demethylmenaquinone-8)
EX_g3pc_e_l	0.0047	8	Sn-Glycero-3-phosphocholine exchange
APRAUR_u	0.00481	6	5-amino-6-(5-phosphoribosylamino)uracil reductase

	original	gdist	Name
ECOAH8_l	0.00483	6	3-hydroxyacyl-CoA dehydratase (3-hydroxyoctadecanoyl-CoA)
PLIPA2A180pp_u	0.00489	6	Phospholipase A2 (phosphatidate, n-C18:0) (periplasm)
GALAMtex_u	0.00491	NA	D Galactosamine transport transport via diffusion extracellular to periplasm
SUCBZS_u	0.005	5	O-succinylbenzoate-CoA synthase
MEOHtex_l	0.00512	7	Methanol transport via diffusion (extracellular to periplasm)
PRMICI_l	0.00513	5	1-(5-phosphoribosyl)-5-[(5-phosphoribosylamino)methylideneamino)imidazole-4-carboxamide isomerase
ACACtex_l	0.00517	6	Acetoacetate transport via diffusion (extracellular to periplasm)
MTHFC_l	0.00519	4	Methenyltetrahydrofolate cyclohydrolase
ACONTa_u	0.0052	5	Aconitase (half-reaction A, Citrate hydro-lyase)
HCYSMT2_u	0.00524	4	Homocysteine Methyltransferase
EX_gam6p_e_u	0.00531	7	D-Glucosamine 6-phosphate exchange
EX_dad_2_e_l	0.00538	8	Deoxyadenosine exchange
EX_glcr_e_l	0.00551	9	D-Glucarate exchange
EX_rmn_e_u	0.0057	10	L-Rhamnose exchange
LPLIPAL2A161_u	0.00587	5	Lysophospholipase L2 (2-acylglycerophosphotidate, n-C16:1)
LEUt2rpp_l	0.00601	4	L-leucine reversible transport via proton symport (periplasm)
ALAGLUE_u	0.00624	5	L-alanyl-gamma-glutamate epimerase
LADGMDH_u	0.00681	4	L-alanyl-gamma-D-glutamyl-meso-diaminopimelate hydrolase
PSCLYSt2pp_u	0.00692	6	Psicoselysine transport via proton symport (periplasm)
EX_psclys_e_l	0.00718	8	Psicoselysine exchange
MDH2_u	0.0072	5	Malate dehydrogenase (ubiquinone 8 as acceptor)
PSCLYStex_u	0.00745	7	Psicoselysine transporter via diffusion (extracellular)
LALGP_u	0.00761	4	L-alanyl-gamma-L-glutamate peptidase
EX_akg_e_l	0.00809	7	2-Oxoglutarate exchange
GRTT_u	0.0084	5	Geranyltranstransferase
EX_3ump_e_u	0.00874	9	3'-UMP exchange
ADNCYC_u	0.00915	NA	Adenylate cyclase
FRULYStex_u	0.00917	6	Fructoselysine transporter via diffusion (extracellular)
DAPabcpp_u	0.00945	4	M-diaminopimelic acid ABC transport (periplasm)
AGM3PH_u	0.00958	6	N-Acetyl-D-glucosamine(anhydrous)N-Acetylmuramyl-tripeptide beta -1,4-N-acetylglucosaminidase
FEOXAMabcpp_u	0.00985	5	Ferroxamine transport via ABC system (periplasm)
SELNPS_u	0.0101	6	Selenophosphate synthase
26DAHtex_u	0.0102	5	Meso-2,6-Diaminoheptanedioate transport via diffusion (extracellular to periplasm)
FRULYSt2pp_u	0.0104	5	Fructoselysine transport via proton symport (periplasm)
**EX_26dap__M_e_u**	0.0112	6	Meso-2,6-Diaminoheptanedioate exchange
EX_frulys_e_l	0.0112	7	Fructoselysine exchange
MOHMT_u	0.0129	4	3-methyl-2-oxobutanoate hydroxymethyltransferase
CBMD_u	0.0172	8	Carbamate deaminase
FRULYSDG_u	0.0498	3	Fructoselysine phosphate deglycase
FRULYSK_u	0.052	4	Fructoselysine Kinase
DAPDC_u	0.12	3	Diaminopimelate decarboxylase
**EX_lys__L_e_u**	0.487	0	L-Lysine exchange
LYStex_l	0.487	1	L-lysine transport via diffusion (extracellular to periplasm)
LYSt3pp_u	0.487	2	L-lysine transport out via proton antiport (cytoplasm to periplasm)

```
pander(df005[order(df005$original),c("original","gdist","Equation")],
      justify = c('left', 'center', 'center', 'left'))
```

	original	gdist	Equation
HACD2i_u	-0.0184	5	3hhcoa_c + nad_c –> 3ohcoa_c + h_c + nadh_c
EX_lcts_e_u	-0.00661	7	lcts_e –>
NOVBCNtpp_u	-0.00624	NA	h_p + novbcn_p –> h_c + novbcn_e
EX_fe3hox_e_l	-0.00595	7	fe3hox_e –>
HACD1_l	-0.00591	5	aacoa_c + h_c + nadh_c <=> 3hbcoa_c + nad_c
4PCPpp_u	-0.00582	5	LalaDgluMdapDala_p + h2o_p –> LalaDgluMdap_p + ala__D_p
NO2t2rpp_l	-0.00559	7	h_p + no2_p <=> h_c + no2_c
FUSAtex_l	-0.00547	NA	fusa_e <=> fusa_p
ACALDtex_l	-0.00545	6	acald_e <=> acald_p
CTBTt2rpp_l	-0.00541	NA	ctbt_p + h_p <=> ctbt_c + h_c
NO3tex_u	-0.00539	7	no3_e <=> no3_p
ARGDCpp_u	-0.00538	5	arg__L_p + h_p –> agm_p + co2_p
EX_pyr_e_l	-0.00535	7	pyr_e –>
CPGNabcpp_u	-0.00525	5	atp_c + cpgn_p + h2o_c –> adp_c + cpgn_c + h_c + pi_c
CFAS180E_u	-0.00517	4	2.0 amet_c + pe181_c –> 2.0 ahcys_c + cpe180_c + 2.0 h_c
A5PISO_l	-0.00505	6	ru5p__D_c <=> ara5p_c
CU2tex_u	-0.00504	5	cu2_e <=> cu2_p
GLUNpp_u	-0.00497	5	gln__L_p + h2o_p –> glu__L_p + nh4_p
MDRPD_u	-0.00489	6	5mdru1p_c –> dkmpp_c + h2o_c
PROt2rpp_l	-0.00487	4	h_p + pro__L_p <=> h_c + pro__L_c
EX_3hpp_e_u	-0.00486	13	3hpp_e –>
**EX_hom__L_e_l**	-0.00482	7	hom__L_e –>
EX_damp_e_l	-0.00482	9	damp_e –>
FACOAE161_u	-0.00481	5	h2o_c + hdcoa_c –> coa_c + h_c + hdcea_c
AACPS9_u	-0.00479	6	ACP_c + atp_c + octa_c –> amp_c + ocACP_c + ppi_c
HPPPNtex_u	-0.00478	9	3hpppn_e <=> 3hpppn_p
GLTPD_l	-0.00469	6	galt1p_c + nad_c <=> h_c + nadh_c + tag6p__D_c
HEPTA1_u	-0.00464	7	adphep_LD_c + lipaA_c –> adp_c + h_c + hlipaA_c
3hoxpactex_l	-0.00459	NA	3hoxpac_e <=> 3hoxpac_p
UDPGtex_l	-0.00458	7	udpg_e <=> udpg_p
MCPST_u	-0.00455	5	cyan_c + mercppyr_c –> h_c + pyr_c + tcynt_c
GLYC2Ptex_u	-0.00454	6	glyc2p_e <=> glyc2p_p
NTD9_u	-0.00453	5	gmp_c + h2o_c –> gsn_c + pi_c
PGI_l	-0.00453	4	g6p_c <=> f6p_c
CBLAT_l	-0.00452	4	atp_c + cbl1_c + h_c <=> adocbl_c + pppi_c
UM4PCP_u	-0.00445	5	h2o_c + um4p_c –> ala__D_c + ugmd_c
HCINNMt2rpp_u	-0.00442	8	3hcinnm_p + h_p <=> 3hcinnm_c + h_c

	original	gdist	Equation
GLUABUTt7pp_u	-0.00439	4	4abut_c + glu__L_p <=> 4abut_p + glu__L_c
M1PD_u	-0.00434	5	mnl1p_c + nad_c <=> f6p_c + h_c + nadh_c
PLIPA2A120pp_u	-0.00433	6	h2o_p + pa120_p –> 1ddecg3p_p + ddca_p + h_p
ACLS_u	-0.00428	5	h_c + 2.0 pyr_c –> alac__S_c + co2_c
EX_cynt_e_l	-0.00421	8	cynt_e –>
2AGPG161tipp_u	-0.0042	5	2agpg161_p –> 2agpg161_c
MALDDH_u	-0.00419	5	mal__D_c + nad_c –> co2_c + nadh_c + pyr_c
GLCURt2rpp_u	-0.00416	9	glcur_p + h_p <=> glcur_c + h_c
AMAOTr_l	-0.00411	4	8aonn_c + amet_c <=> amob_c + dann_c
ASPK_l	-0.00404	4	asp__L_c + atp_c <=> 4pasp_c + adp_c
SERtex_l	-0.00404	5	ser__L_e <=> ser__L_p
MOX_u	-0.00402	5	mal__L_c + o2_c <=> h2o2_c + oaa_c
ASO3tex_l	-0.004	9	aso3_e <=> aso3_p
FALDH2_l	-0.00396	6	hmgth_c + nad_c <=> Sfglutth_c + h_c + nadh_c
GLCNt2rpp_u	-0.00392	6	glcn_p + h_p <=> glcn_c + h_c
ANS_u	-0.00392	4	chor_c + gln__L_c –> anth_c + glu__L_c + h_c + pyr_c
NAtex_l	-0.00392	NA	NA
FCLPA_l	-0.00388	5	fc1p_c <=> dhap_c + lald__L_c
ME1_u	-0.00388	5	mal__L_c + nad_c –> co2_c + nadh_c + pyr_c
PRPPS_l	-0.00386	5	atp_c + r5p_c <=> amp_c + h_c + prpp_c
UREAtpp_l	0.00386	5	urea_p <=> urea_c
EX_r5p_e_u	0.00388	9	r5p_e –>
ALAALAr_u	0.00391	5	2.0 ala__D_c + atp_c <=> adp_c + alaala_c + h_c + pi_c
FEOXAMUtex_u	0.00393	6	feoxam_un_p + h_p –> feoxam_un_e + h_c
PUNP6_l	0.00402	6	din_c + pi_c <=> 2dr1p_c + hxan_c
NDPK3_l	0.00404	4	atp_c + cdp_c <=> adp_c + ctp_c
CLPNS141pp_l	0.00407	5	2.0 pg141_p <=> clpn141_p + glyc_p
EX_23ccmp_e_l	0.00408	10	23ccmp_e –>
NDPK7_l	0.00411	4	atp_c + dcdp_c <=> adp_c + dctp_c
EX_glyald_e_l	0.00414	9	glyald_e –>
NO3R2bpp_u	0.00415	7	mql8_c + no3_p –> h2o_p + mqn8_c + no2_p
F6PA_l	0.00421	5	f6p_c <=> dha_c + g3p_c
SPMDabcpp_u	0.00424	4	atp_c + h2o_c + spmd_p –> adp_c + h_c + pi_c + spmd_c
FRULYStex_l	0.00426	6	frulys_e <=> frulys_p
EX_slnt_e_l	0.00431	10	slnt_e <=>
GLYC2Ptex_l	0.00431	6	glyc2p_e <=> glyc2p_p
EX_acgam1p_e_u	0.00431	8	acgam1p_e –>
DINStex_l	0.00432	8	din_e <=> din_p

	original	gdist	Equation
SUCBZL_u	0.00435	6	atp_c + coa_c + sucbz_c -> amp_c + ppi_c + sbzcoa_c
EX_ocdca_e_l	0.00443	8	ocdca_e ->
EX_cl_e_l	0.00446	6	cl_e <=>
EX_hg2_e_l	0.00449	NA	hg2_e ->
PTAr_u	0.00451	4	accoa_c + pi_c <=> actp_c + coa_c
DALAtex_l	0.0046	5	ala__D_e <=> ala__D_p
EX_g3pe_e_l	0.00462	7	g3pe_e ->
G3PD7_u	0.00463	4	2dmmq8_c + glyc3p_c -> 2dmmql8_c + dhap_c
EX_g3pc_e_l	0.0047	8	g3pc_e ->
APRAUR_u	0.00481	6	5apru_c + h_c + nadph_c -> 5aprbu_c + nadp_c
ECOAH8_l	0.00483	6	3hodcoa_c <=> h2o_c + od2coa_c
PLIPA2A180pp_u	0.00489	6	h2o_p + pa180_p -> 1odecg3p_p + h_p + ocdca_p
GALAMtex_u	0.00491	NA	galam_e <=> galam_p
SUCBZS_u	0.005	5	2shchc_c -> h2o_c + sucbz_c
MEOHtex_l	0.00512	7	meoh_e <=> meoh_p
PRMICI_l	0.00513	5	prfp_c <=> prlp_c
ACACtex_l	0.00517	6	acac_e <=> acac_p
MTHFC_l	0.00519	4	h2o_c + methf_c <=> 10fthf_c + h_c
ACONTa_u	0.0052	5	cit_c <=> acon_C_c + h2o_c
HCYSMT2_u	0.00524	4	hcys__L_c + mmet_c -> h_c + 2.0 met__L_c
EX_gam6p_e_u	0.00531	7	gam6p_e ->
EX_dad_2_e_l	0.00538	8	dad_2_e ->
EX_glcr_e_l	0.00551	9	glcr_e ->
EX_rmn_e_u	0.0057	10	rmn_e ->
LPLIPAL2A161_u	0.00587	5	2hdec9eg3p_c + h2o_c -> glyc3p_c + 2.0 h_c + hdcea_c
LEUt2rpp_l	0.00601	4	h_p + leu__L_p <=> h_c + leu__L_c
ALAGLUE_u	0.00624	5	LalaDglu_c <=> LalaLglu_c
LADGMDH_u	0.00681	4	LalaDgluMdap_c + h2o_c -> 26dap__M_c + LalaDglu_c
PSCLYSt2pp_u	0.00692	6	h_p + psclys_p -> h_c + psclys_c
EX_psclys_e_l	0.00718	8	psclys_e ->
MDH2_u	0.0072	5	mal__L_c + q8_c -> oaa_c + q8h2_c
PSCLYStex_u	0.00745	7	psclys_e <=> psclys_p
LALGP_u	0.00761	4	LalaLglu_c + h2o_c -> ala__L_c + glu__L_c
EX_akg_e_l	0.00809	7	akg_e ->
GRTT_u	0.0084	5	grdp_c + ipdp_c -> frdp_c + ppi_c
EX_3ump_e_u	0.00874	9	3ump_e ->
ADNCYC_u	0.00915	NA	atp_c -> camp_c + ppi_c
FRULYStex_u	0.00917	6	frulys_e <=> frulys_p
DAPabcpp_u	0.00945	4	26dap__M_p + atp_c + h2o_c -> 26dap__M_c + adp_c + h_c + pi_c
AGM3PH_u	0.00958	6	anhgm3p_c + h2o_c -> acgam_c + anhm3p_c
FEOXAMabcpp_u	0.00985	5	atp_c + feoxam_p + h2o_c -> adp_c + feoxam_c + h_c + pi_c

	original	gdist	Equation
SELNPS_u	0.0101	6	atp_c + h2o_c + seln_c -> amp_c + pi_c + selnp_c
26DAHtex_u	0.0102	5	26dap__M_e <=> 26dap__M_p
FRULYSt2pp_u	0.0104	5	frulys_p + h_p -> frulys_c + h_c
**EX_26dap__M_e_l**	0.0112	6	26dap__M_e ->
EX_frulys_e_l	0.0112	7	frulys_e ->
MOHMT_u	0.0129	4	3mob_c + h2o_c + mlthf_c -> 2dhp_c + thf_c
CBMD_u	0.0172	8	cbm_c + 2.0 h_c -> co2_c + nh4_c
FRULYSDG_u	0.0498	3	frulysp_c + h2o_c <=> g6p_c + lys__L_c
FRULYSK_u	0.052	4	atp_c + frulys_c -> adp_c + frulysp_c + h_c
DAPDC_u	0.12	3	26dap__M_c + h_c -> co2_c + lys__L_c
**EX_lys__L_e_u**	0.487	0	lys__L_e ->
LYStex_l	0.487	1	lys__L_e <=> lys__L_p
LYSt3pp_u	0.487	2	h_p + lys__L_c -> h_c + lys__L_p

2 Appendix

2.1 Functions

```
## Custom functions used in the analysis should go into this chunk.
## They will be listed in their own section of the appendix.

##==================== Functions ====================##
```

2.2 Setup R

```
## This chunk should contain global configuration commands.
## Use this to set knitr options and related things. Everything
## in this chunk will be included in an appendix to document the
## configuration used.
#output <- opts_knit$get("rmarkdown.pandoc.to")
knitr::opts_knit$set(stop_on_error = 2L)

## By default R code is only included in HTML versions of the report
## (where it can be collapsed). You can generate a PDF version
## using rmarkdown::pdf_document to get a copy for print. Extensive
## chunks of R code may or may not be desired in /hat setting. If you
## want them simply change the following arguments to `echo = TRUE`.
## In either case the default can be overwritten for individual chunks.
#opts_chunk$set(echo = output=="html")
#opts_chunk$set(warning = output=="html")
#opts_chunk$set(message = output=="html")

## Cache options
knitr::opts_chunk$set(cache=TRUE,warning=FALSE)
```

```
## Figure options
## Set default figure format
#options(reportmd.figure.format=params$format)

## Set 'hide.fig.code' to FALSE to include code chunks that
## produce Figures in the output. Note that this affects all chunks
## that provide a figure caption.
knitr::opts_chunk$set(hold=TRUE, hide.fig.code=FALSE)

## Set up default plotting options for different formats.
## These can be overwritten for individual chunks
#interactiveFig()
#screenFig()
#printFig()

## Pander options
pander::panderOptions("digits", 3)
pander::panderOptions("table.split.table", 160)
#panderOptions("table.style", "grid")

## Configure Figure and Table lables
#options(figcap.prefix = "Figure", figcap.sep = ":", figcap.prefix.highlight = "**")
#options(tabcap.prefix = "Table", tabcap.sep = ":", tabcap.prefix.highlight = "**")

## Install required knitr hooks
#installHooks()
```

2.3 Versions

2.3.1 Document version

2.3.2 Session Info

Platform

- version: R version 4.2.1 (2022-06-23)
- os: macOS Big Sur … 10.16
- system: x86_64, darwin17.0
- ui: X11
- language: (EN)
- collate: en_US.UTF-8
- ctype: en_US.UTF-8
- tz: Asia/Tokyo
- date: 2022-12-04
- pandoc: 2.19.2 @ /Applications/RStudio.app/Contents/MacOS/quarto/bin/tools/ (via rmarkdown)

Packages

	ondiskversion	loadedversion	date	source
assertthat	0.2.1	0.2.1	2019-03-21	CRAN (R 4.2.0)
backports	1.4.1	1.4.1	2021-12-13	CRAN (R 4.2.0)
boot	1.3.28	1.3-28	2021-05-03	CRAN (R 4.2.1)
cachem	1.0.6	1.0.6	2021-08-19	CRAN (R 4.2.0)
callr	3.7.3	3.7.3	2022-11-02	CRAN (R 4.2.0)
checkmate	2.1.0	2.1.0	2022-04-21	CRAN (R 4.2.0)

	ondiskversion	loadedversion	date	source
class	7.3.20	7.3-20	2022-01-16	CRAN (R 4.2.1)
cli	3.4.1	3.4.1	2022-09-23	CRAN (R 4.2.0)
clue	0.3.62	0.3-62	2022-10-18	CRAN (R 4.2.0)
cluster	2.1.4	2.1.4	2022-08-22	CRAN (R 4.2.0)
codetools	0.2.18	0.2-18	2020-11-04	CRAN (R 4.2.1)
colorspace	2.0.3	2.0-3	2022-02-21	CRAN (R 4.2.0)
crayon	1.5.2	1.5.2	2022-09-29	CRAN (R 4.2.0)
data.table	1.14.4	1.14.4	2022-10-17	CRAN (R 4.2.1)
DBI	1.1.3	1.1.3	2022-06-18	CRAN (R 4.2.0)
deSolve	1.34	1.34	2022-10-22	CRAN (R 4.2.0)
devtools	2.4.5	2.4.5	2022-10-11	CRAN (R 4.2.1)
digest	0.6.30	0.6.30	2022-10-18	CRAN (R 4.2.0)
dplyr	1.0.10	1.0.10	2022-09-01	CRAN (R 4.2.1)
dtw	1.23.1	1.23-1	2022-09-19	CRAN (R 4.2.0)
dtwclust	5.5.11	5.5.11	2022-09-24	CRAN (R 4.2.0)
ellipsis	0.3.2	0.3.2	2021-04-29	CRAN (R 4.2.0)
evaluate	0.18	0.18	2022-11-07	CRAN (R 4.2.0)
fansi	1.0.3	1.0.3	2022-03-24	CRAN (R 4.2.0)
farver	2.1.1	2.1.1	2022-07-06	CRAN (R 4.2.0)
fastmap	1.1.0	1.1.0	2021-01-25	CRAN (R 4.2.0)
flexclust	1.4.1	1.4-1	2022-04-08	CRAN (R 4.2.0)
foreach	1.5.2	1.5.2	2022-02-02	CRAN (R 4.2.0)
fs	1.5.2	1.5.2	2021-12-08	CRAN (R 4.2.0)
generics	0.1.3	0.1.3	2022-07-05	CRAN (R 4.2.0)
ggplot2	3.4.0	3.4.0	2022-11-04	CRAN (R 4.2.0)
ggrepel	0.9.2	0.9.2	2022-11-06	CRAN (R 4.2.0)
glue	1.6.2	1.6.2	2022-02-24	CRAN (R 4.2.0)
gtable	0.3.1	0.3.1	2022-09-01	CRAN (R 4.2.1)
highr	0.9	0.9	2021-04-16	CRAN (R 4.2.0)
htmltools	0.5.3	0.5.3	2022-07-18	CRAN (R 4.2.0)
htmlwidgets	1.5.4	1.5.4	2021-09-08	CRAN (R 4.2.0)
httpuv	1.6.6	1.6.6	2022-09-08	CRAN (R 4.2.0)
iterators	1.0.14	1.0.14	2022-02-05	CRAN (R 4.2.0)
knitr	1.41	1.41	2022-11-18	CRAN (R 4.2.0)
labeling	0.4.2	0.4.2	2020-10-20	CRAN (R 4.2.0)
later	1.3.0	1.3.0	2021-08-18	CRAN (R 4.2.0)
lattice	0.20.45	0.20-45	2021-09-22	CRAN (R 4.2.1)
lifecycle	1.0.3	1.0.3	2022-10-07	CRAN (R 4.2.1)
magrittr	2.0.3	2.0.3	2022-03-30	CRAN (R 4.2.0)
Matrix	1.5.1	1.5-1	2022-09-13	CRAN (R 4.2.0)
memoise	2.0.1	2.0.1	2021-11-26	CRAN (R 4.2.0)
mime	0.12	0.12	2021-09-28	CRAN (R 4.2.0)
miniUI	0.1.1.1	0.1.1.1	2018-05-18	CRAN (R 4.2.0)
modeltools	0.2.23	0.2-23	2020-03-05	CRAN (R 4.2.0)
munsell	0.5.0	0.5.0	2018-06-12	CRAN (R 4.2.0)
numbers	0.8.2	0.8-2	2021-05-14	CRAN (R 4.2.0)
ODEnetwork	1.3.2	1.3.2	2020-04-03	CRAN (R 4.2.0)
ODEsensitivity	1.1.2	1.1.2	2019-01-09	CRAN (R 4.2.0)
openxlsx	4.2.5.1	4.2.5.1	2022-10-24	CRAN (R 4.2.0)
pander	0.6.5	0.6.5	2022-03-18	CRAN (R 4.2.0)
pillar	1.8.1	1.8.1	2022-08-19	CRAN (R 4.2.0)
pkgbuild	1.3.1	1.3.1	2021-12-20	CRAN (R 4.2.0)

	ondiskversion	loadedversion	date	source
pkgconfig	2.0.3	2.0.3	2019-09-22	CRAN (R 4.2.0)
pkgload	1.3.1	1.3.1	2022-10-28	CRAN (R 4.2.0)
plyr	1.8.8	1.8.8	2022-11-11	CRAN (R 4.2.0)
prettyunits	1.1.1	1.1.1	2020-01-24	CRAN (R 4.2.0)
processx	3.8.0	3.8.0	2022-10-26	CRAN (R 4.2.0)
profvis	0.3.7	0.3.7	2020-11-02	CRAN (R 4.2.0)
promises	1.2.0.1	1.2.0.1	2021-02-11	CRAN (R 4.2.0)
proxy	0.4.27	0.4-27	2022-06-09	CRAN (R 4.2.0)
ps	1.7.2	1.7.2	2022-10-26	CRAN (R 4.2.0)
purrr	0.3.5	0.3.5	2022-10-06	CRAN (R 4.2.1)
R6	2.5.1	2.5.1	2021-08-19	CRAN (R 4.2.0)
Rcpp	1.0.9	1.0.9	2022-07-08	CRAN (R 4.2.0)
RcppParallel	5.1.5	5.1.5	2022-01-05	CRAN (R 4.2.0)
remotes	2.4.2	2.4.2	2021-11-30	CRAN (R 4.2.0)
reshape2	1.4.4	1.4.4	2020-04-09	CRAN (R 4.2.0)
rlang	1.0.6	1.0.6	2022-09-24	CRAN (R 4.2.0)
rmarkdown	2.18	2.18	2022-11-09	CRAN (R 4.2.1)
RSpectra	0.16.1	0.16-1	2022-04-24	CRAN (R 4.2.0)
rstudioapi	0.14	0.14	2022-08-22	CRAN (R 4.2.0)
scales	1.2.1	1.2.1	2022-08-20	CRAN (R 4.2.0)
sensitivity	1.28.0	1.28.0	2022-09-29	CRAN (R 4.2.0)
sessioninfo	1.2.2	1.2.2	2021-12-06	CRAN (R 4.2.0)
shiny	1.7.3	1.7.3	2022-10-25	CRAN (R 4.2.0)
shinyjs	2.1.0	2.1.0	2021-12-23	CRAN (R 4.2.0)
stringi	1.7.8	1.7.8	2022-07-11	CRAN (R 4.2.0)
stringr	1.4.1	1.4.1	2022-08-20	CRAN (R 4.2.1)
tibble	3.1.8	3.1.8	2022-07-22	CRAN (R 4.2.0)
tidyr	1.2.1	1.2.1	2022-09-08	CRAN (R 4.2.1)
tidyselect	1.2.0	1.2.0	2022-10-10	CRAN (R 4.2.1)
urlchecker	1.0.1	1.0.1	2021-11-30	CRAN (R 4.2.0)
usethis	2.1.6	2.1.6	2022-05-25	CRAN (R 4.2.0)
utf8	1.2.2	1.2.2	2021-07-24	CRAN (R 4.2.0)
vctrs	0.5.1	0.5.1	2022-11-16	CRAN (R 4.2.0)
withr	2.5.0	2.5.0	2022-03-03	CRAN (R 4.2.0)
xfun	0.35	0.35	2022-11-16	CRAN (R 4.2.0)
xtable	1.8.4	1.8-4	2019-04-21	CRAN (R 4.2.0)
yaml	2.3.6	2.3.6	2022-10-18	CRAN (R 4.2.0)
zip	2.2.2	2.2.2	2022-10-26	CRAN (R 4.2.0)
